# The function of LncRNA-H19 in cardiac hypertrophy

**DOI:** 10.1186/s13578-021-00668-4

**Published:** 2021-08-03

**Authors:** Wenhua Su, Qian Huo, Hao Wu, Lulin Wang, Xiaoxue Ding, Liwen Liang, Liang Zhou, Yan Zhao, Juhua Dan, Hong Zhang

**Affiliations:** 1 Faculty of Life Science and Biotechnology, Kunming University of Science and Technology, 650500 Kunming, People’s Republic of China; 2 Department of Cardiology, The First People’s Hospital of Yunnan Province, 650032 Kunming, People’s Republic of China

**Keywords:** Cardiovascular hypertrophy, LncRNA-H19, Cardiomyocyte remodeling, Therapy

## Abstract

Cardiac hypertrophy, characterized by the enlargement of cardiomyocytes, is initially an adaptive response to physiological and pathological stimuli. Decompensated cardiac hypertrophy is related to fibrosis, inflammatory cytokine, maladaptive remodeling, and heart failure. Although pathological myocardial hypertrophy is the main cause of hypertrophy-related morbidity and mortality, our understanding of its mechanism is still poor. Long noncoding RNAs (lncRNAs) are noncoding RNAs that regulate various physiological and pathological processes through multiple molecular mechanisms. Recently, accumulating evidence has indicated that lncRNA-H19 is a potent regulator of the progression of cardiac hypertrophy. For the first time, this review summarizes the current studies about the role of lncRNA-H19 in cardiac hypertrophy, including its pathophysiological processes and underlying pathological mechanism, including calcium regulation, fibrosis, apoptosis, angiogenesis, inflammation, and methylation. The context within which lncRNA-H19 might be developed as a target for cardiac hypertrophy treatment is then discussed to gain better insight into the possible biological functions of lncRNA-H19 in cardiac hypertrophy.

## Introduction

Hypertrophic cardiomyopathy (HCM) is a common inherited disease characterized by an increase in the thickness of the ventricular wall (≥ 1.5 cm) in the absence of increased afterload, and it is recognized as an important cause of sudden cardiac death among young adults and competitive athletes [1]. In recent years, abundant data have revealed that HCM occurs at a rate of approximately 1/500 in the general population [2], but other data indicate a prevalence of HCM and genetic carriers of 1/200 [3]. Cardiac hypertrophy (CH), characterized by an increase in cardiomyocyte size, rather than an increase in their number, is initially a compensatory response to cope with biomechanical stresses and facilitate the maintenance of proper cardiac output and homeostasis [4, 5].

CH has been categorized as either pathological or physiological hypertrophy. Physiological CH is generally caused by normal growth, pregnancy, or exercise. Pathological CH is the heart’s maladaptive reaction to various pathological stimuli, such as high blood pressure, myocardial infarction, and many more [6]. Pathological CH is related to myocardial fibrosis, calcium (Ca^2+^) dysregulation, increased inflammatory cytokine, and epigenetic changes, which lead to maladaptive cardiac remodeling, heart failure (HF), and death [7, 8]. The progression of both physiological and pathological hypertrophy depends on upstream stimuli and signaling mechanisms rather than cardiac stress [9–11]. The current efficiency of CH treatment is improving rapidly, but the mortality rate of HF remains at approximately 50% 5 years after diagnosis [12]. Therefore, identifying the fundamental molecular mechanisms underlying CH is a vital challenge for HF treatment.

In the past decade, numerous studies have found that some regulatory mechanisms, including cellular metabolism [13], proliferation [6], miRNAs [14–16], immune responses [17, 18], translational regulation [19], and epigenetic modifications [20, 21], positively or negatively regulate CH. Global transcriptome analyses have identified a few lncRNAs that are critically involved in CH [22–24].

Recently, long noncoding RNAs (lncRNAs), which are more than 200 nucleotides in length and lack protein-coding capacity [25], have been shown to be involved in many cellular processes and the development of various diseases [26, 27]. By interacting with RNA, DNA and proteins, lncRNAs can regulate the expression of genes involved in many biological activities [28, 29], such as RNA processing [30], apoptosis [31], genome rearrangement and chromatin modification [32, 33], and competing endogenous RNAs (ceRNAs) [34, 35], at multiple levels. Therefore, lncRNAs are dynamically expressed in a range of differentiation processes, including those of embryonic stem cells [36, 37], vascular smooth muscle cells [38], muscles [39], T cells [40], breast tissues [41] and neurons [42], as well as in cancer [42, 43] and other diseases [44, 45]. Moreover, many studies have shown that lncRNAs are important regulators in many pathophysiological processes of heart development and diseases [22, 46, 47], such as cardiac organogenesis [48], atherosclerosis [49–51], hypertension [38, 52, 53], pulmonary arterial hypertension [54], coronary artery disease [55, 56], ischemia/reperfusion-induced apoptosis [57], HF [58], and CH (Table 1). All these results suggest that lncRNAs play a central role in the occurrence of human diseases, including cardiovascular diseases.

**Table 1:** Anti- and pro-hypertrophic lncRNAs and their regulated genes

LncRNA	Regulated genes	References
Anti-hypertrophic lncRNAs		
Plscr4	miR-214‐Mfn2	Lv et al. [59]
Ahit	SUZ12/PRC2-MEF2A	Yu et al. [60]
Uc.323	EZH2-CPT1b	Sun et al. [61]
TINCR	EZH2- CaMKII	Cai et al. [62]
XIST	miR-330-3p/S100B	Chen et al. [63]
SNHG1	miR-15a-5p/HMGA1	Yan et al. [64]
Mhrt	miR-145a‐5p/KLF4/myocardin	Xu et al. [65]
Mhrt	Brg1	Han et al. [66]
H19	miR-675/CaMKIId	Liu et al. [67]
H19	PCR2- NFAT	Viereck et al. [68]
HOTAIR	miRi19/PTEN	Lai et al. [69]
MAGI1-IT1	miR-302e/DKK1/Wnt/β-catenin	Zhang et al. [70]
TUG1	miR-29b-3p	Zou et al. [71]
Kcnq1ot1	miR-30e-5p/ADAM9	Wang et al. [72]
AK045171	SP1/MG53	Xu et al. [73]
Pro-hypertrophic lncRNAs		
Chaer	PRC2	Wang et al. [21]
Chast	Plekhm1	Viereck et al. [74]
MEG3	miR-361-5p/HDAC9	Zhang et al. [75]
DACH1	SERCA2a	Frey et al. [76]
XIST	miR-101/TLR2	Xiao et al. [77]
CHRF	miR-489/ ;Myd88/NF-κB	Wang et al. [78]
CHRF	miR-93/Akt3	Wo et al. [79]
SYNE1-AS1	miR-525-5p/SP1	Wang et al. [80]
CASC15	miR-432-5p/TLR4 ;axis	Li et al. [81]
MIAT	miR1505p/P300	Li et al. [82]
SNHG14	miR-322‐5p/miR‐384‐5p/PCDH17	Long et al. [83]
SNHG16	miR-182-5p/IGF1	Wang et al. [84]
CASC15	miR-432-5p/TLR4	Li et al. [81]
PEG10	PEG10	Wen et al. [85]
ROR	miR-133	Jiang et al. [86]

The evidence described above indicates that a large number of lncRNAs are positively or negatively correlated with CH. These lncRNAs participate in complex networks in the pathological process of CH by interacting with contractile protein expression, calcium processing, and mitochondrial function. Among these lncRNAs, lncRNA H19 (hereafter called H19) has attracted our great interest

H19, which is a maternally expressed and paternally imprinted 2.7-kb gene, is localized near the telomeric region of chromosome 11p15.5 and is reciprocally imprinted and regulated with its neighboring gene, insulin-like growth factor 2 (IGF2) [28, 87, 88]. H19 is highly evolutionarily conserved, suggesting that it may have some crucial biological functions [89]. Intriguingly, H19 is most highly expressed in skeletal muscle and exhibits an ~ tenfold enrichment in cardiac tissue over all other mouse tissues (such as brain, lung, kidney, and many more) [68].

Emerging evidence shows that diverse cells, such as hematopoietic stem cells [90] and neurons [91, 92], and cellular processes, including abdominal aortic aneurysm [93], diabetic nephropathy [94], hepatocyte proliferation [95, 96], tumorigenesis [97, 98], acute promyelocytic leukemia [99], ulcerative colitis [100], senescence [101], and endometriosis [102], are regulated by H19. Moreover, accumulating evidence indicates that H19 is a powerful regulator of cardiac development and pathophysiology, such as endothelial aging [103], mineralization of aortic valves [104], ischemia/reperfusion injury [105, 106], and atherosclerosis [107]. All these studies show that H19 plays a crucial role in the occurrence and development of heart disease and will become a new hot spot and focus of cardiovascular basic and clinical research.

H19 expression is dynamically regulated, and it is involved in multiple pathways of different cardiac cell types to exert distinct cell type-specific effects. In different stages of CH, H19 expression is different. During the process of heart maturation after birth and with age, the expression of H19 gradually decreases [67] but increases within 2 weeks after transverse aortic constriction (TAC, a surgical procedure that induces CH) in a mouse model [67, 108]. Nonetheless, H19 expression was significantly decreased during the progression from the compensated stage to the decompensated stage of HF (4–6 weeks after TAC) and remained low until the experimental endpoint 13 weeks after TAC [68]. Of note, due to differences in mean age between patients with diseased and healthy hearts, lower levels of H19 expression in human heart tissues may be partially attributed to an age-related reduction.

**Fig. 1:**
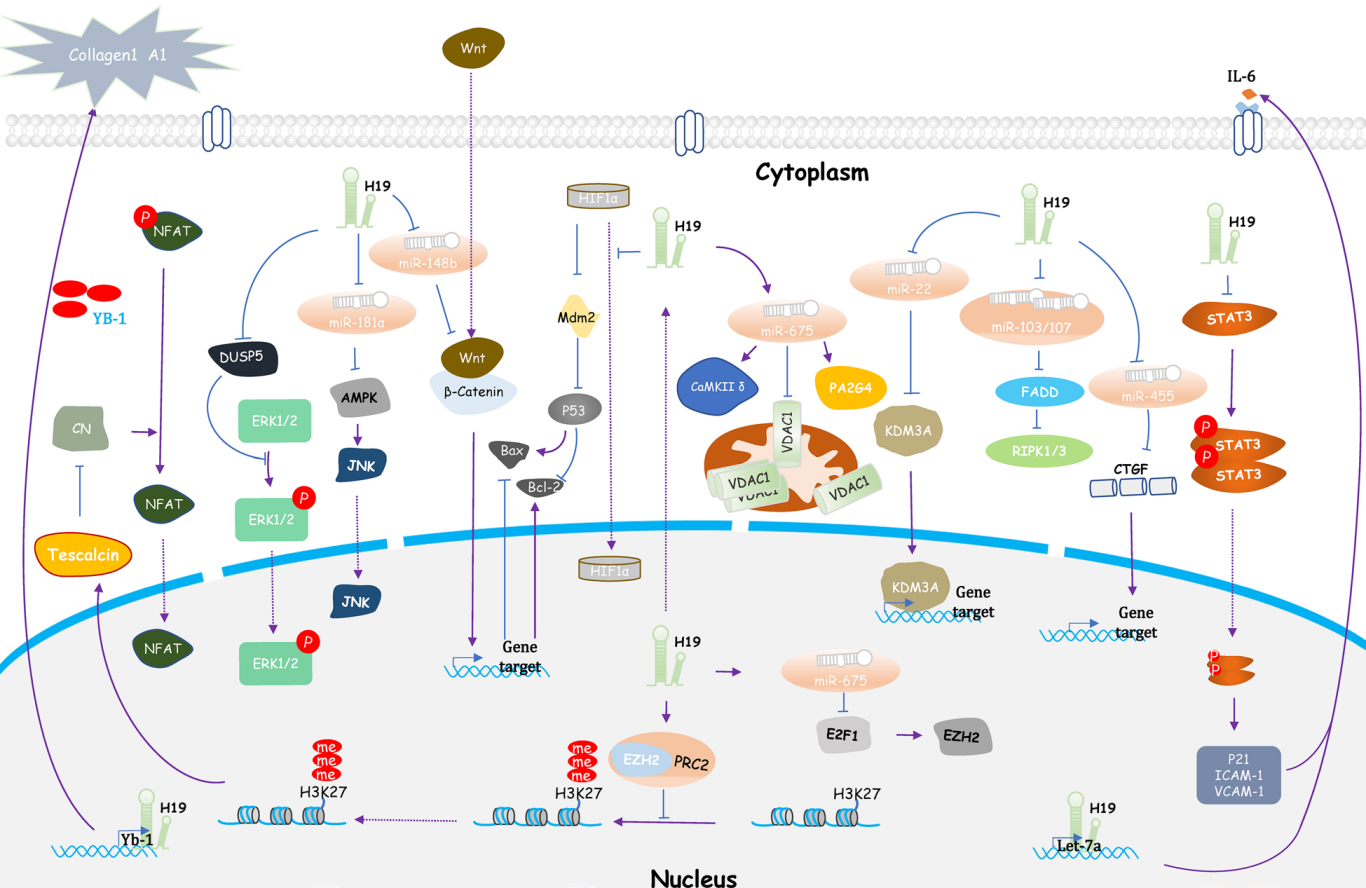
The role of H19 and its targets in CH-related pathophysiological processes and the potential mechanisms. *AMPK* adenosine 5′-monophosphate (AMP)-activated protein kinase, *CaMKIIδ* Ca^2+^/calmodulin-dependent protein kinase IIδ, *CN* calcineurin, *CTGF* connective tissue growth factor, *DUSP5* dual-specificity phosphatase 5, *E2F1* E2F transcription factor 1, *EZH2* enhancer of zeste homolog 2, *FADD* fas-associated protein with death domain, *HIF-1α* hypoxia-inducible factor 1α, *ICAM-1* intercellular adhesion molecule 1, *JNK* jun n-terminal kinases, *KDM3A* Lysine (K)-specific demethylase 3 A, *MDM2* mouse 3T3 cell double minute 2, *NFAT* nuclear factor of activated T cell, *PA2G4* proliferation-associated protein 2G4, *PRC2* polycomb suppression complex 2, *RIPK* receptor-interacting serine/threonine-protein kinase 1 and 3, *STAT3* signal transducer and activator of transcription 3, *VCAM-1* vascular cell adhesion molecule 1, *VDAC1* voltage-dependent anion channel 1, *YB-1* Y-box-binding protein-1

During the pathogenesis of CH, intracellular signals, such as calcium regulation, are regulated to promote the translocation of the hypertrophy-related transcription factor NFAT to regulate the expression of downstream hypertrophy genes. These signals promote myocardial fibrosis and CH. The long-term presence of stress promotes angiogenesis, inflammation, and then apoptosis, ultimately leading to heart failure. Numerous investigations have shown that H19 is involved in some of these pathophysiologies. Herein, we summarized the current studies on H19 in CH-related pathophysiological processes (Fig. 1) to explain the potential therapeutic value of H19 in HCM and provide a basis for further investigation.

## LncRNA-H19 controls the function of calcium channels

Cyclic changes in calcium (Ca^2+^) in cardiomyocytes regulate the contraction of the heart [109, 110]. Ca^2+^/calmodulin-dependent protein kinase IIδ (CaMKIIδ) is associated with the phosphorylation of some Ca^2+^-regulating proteins and has been shown to act as an inducer of CH [111–113]. Moreover, evidence emphasizes that Ca^2+^ dysregulation plays a key role in the development of CH [114].

H19, as the precursor of miR-675, which inhibits the expression of CaMKIIδ, is a negative regulator of CH. Exon 1 of H19 contains a miRNA-containing hairpin, and it has been shown that miR-675 can confer functionality to H19 [115, 116]. The mRNA 3′-UTR of miR-675, a miRNA embedded in H19’s first exon, matches the sequence of CaMKIIδ. Liu et al. revealed that the H19-miR-675-CaMKIIδ axis plays a crucial role in CH. The expression of H19 and miR-675 were found to be upregulated in pathological cardiac hypertrophy, and CaMKIIδ was shown to be a direct target of miR-675 and to partially mediate the effect of H19 on cardiomyocytes, indicating that miR-675-regulated CaMKIIδ might mediate the H19-induced inhibition of cardiomyocyte hypertrophy. Similarly, another study found that CaMKIIδ expression was upregulated after the suppression of H19 expression in a rat right ventricular failure model established by pulmonary artery banding [58]. Nonetheless, Thum, who found that repression or overexpression of H19 had no influence on the expression levels of CaMKIIδ, thought that these antihypertrophic effects are H19-specific and independent of miR-675 [68]. It may be that the expression levels of CaMKIIδ are different at different stages and that many other molecules can activate CaMKIIδ. The levels of CaMKIIδ increase as early as 2 days and continuously for up to 7 days after TAC surgery [117]. In addition, splice variants of the CaMKIIδ isoform, characterized by the presence of a second variable domain, include CaMKII δ_B_ and CaMKIIδ_C_ [118]. Consequently, further research is required to investigate which isoform of CaMKIIδ mediates the H19-induced inhibition of cardiomyocyte hypertrophy.

## LncRNA-H19 regulates transcription factors

Methylation has been implicated as one of the modulators of cardiac gene expression in development and disease [119]. A study revealed that trimethylation of histone H3 at lysine (K) 4, K9, or K27 and dimethylation of H3 at K9 and K79 were associated with hypertrophic heart phenotypes [120]. Different lncRNAs were shown to favor or prevent binding and methylation by interacting with polycomb suppression complex 2 (PRC2) [21, 46], which trimethylates lysine residue 27 on histone H3 [121]. Viereck et al. found that H19 exerts its antihypertrophic function by targeting the prohypertrophic nuclear factor of activated T cell (NFAT) signaling pathway [68]. H19 interacts with PRC2 to suppress H3K27 trimethylation (H3K27me3) of the antihypertrophic Tescalcin locus, resulting in a decrease in the expression and activity of NFAT. Furthermore, in vitro and in vivo, the absence of H19 leads to repression of Tescalcin, which in turn increases NFAT levels and effects on its pro-hypertrophic target genes. These studies suggest that H19-mediated regulation of methylation might alleviate the progression of pathological hypertrophy.

## LncRNA-H19 regulates cardiac fibrosis

Fibrosis is characterized by the net accumulation of extracellular matrix (ECM) proteins and develops because of fibroblast differentiation during the process of inflammation [122]. In a normal heart, cardiac fibroblasts generate ECM components, such as collagen type I and type III. Cardiac fibrosis, which occurs due to the aberrant deposition of ECM proteins in the cardiac interstitium, leads to systolic and/or diastolic dysfunction in many cardiac pathological conditions, including myocardial infarction, cardiomyopathy, and HF, resulting in serious cardiac dysfunction [123].

Studies show that H19 is highly associated with organ fibrosis, including liver fibrosis [96], lung fibrosis [124], renal fibrosis [56], and cardiac fibrosis [125, 126]. Evidence has demonstrated that H19-mediated regulation of DUSP5 affects ERK1/2 phosphorylation, increasing cardiac fibroblast proliferation and fibrosis [127]. Another study revealed that H19 knockdown could enhance the antifibrotic role of miR-455, decrease connective tissue growth factor (CTGF) expression, and further reduce fibrosis-associated protein synthesis [128]. Moreover, in phenylephrine-induced pathological cardiomyocyte hypertrophy, H19 knockdown upregulated the expression of enhancer of zeste homolog 2 (EZH2) [126], which is known to target cardiac myocytes and silence hypertrophic and fibrotic gene programs [129]. Additionally, Choong et al. verified that H19 acted to antagonize Y-box-binding protein-1 (YB-1) through direct interaction under hypoxic conditions, which led to the downregulation of Collagen 1A1 expression and cardiac fibrosis, aggravating cardiac remodeling [125]. The above experimental results suggest that H19 directly or indirectly promotes cardiac fibrosis by acting as a molecular sponge or interacting with various proteins to regulate gene expression.

However, other studies obtained the opposite results. The expression of H19 in cardiomyocytes, among the major cardiac cell types, is lower than that in endothelial cells but higher than that in cardiac fibroblasts, and H19 regulates cardiac fibroblast proliferation and fibrosis [68].

These experiments suggest that H19 may be involved in the different pathological processes of CH through different molecular mechanisms, which has established a solid foundation for the future development of novel treatments for cardiac fibrosis.

## LncRNA-H19 regulates angiogenesis

Angiogenesis, which is induced by paracrine signals between myocardial cells and the vascular system, is a key component of cardiac remodeling [130]. The development of hypertrophy is affected by capillary density. Vascular endothelial growth factor (VEGF) is an essential angiogenic molecule involved in maintaining myocardial capillary density. In pathological hypertrophy, capillary density and coronary blood flow reserve are not enough to support myocardial growth, leading to mild hypoxia and nutritional insufficiency of the myocardium [131].

Research has revealed that H19 is involved in vascular angiogenesis. H19 knockdown led to a dramatic reduction in endothelial cell (EC) growth and formation of a capillary-like structure, which was related to cell cycle inhibition [132]. Additionally, another study showed that the endothelium-specific inhibition of H19 could impair angiogenesis, while exogenous H19 could partially protect this effect [103]. Moreover, Zhu et al. reported that H19 overexpression also increases VEGF protein levels and endothelial NO synthase (eNOS) levels in human dermal vascular endothelial cells (HMEC-1) by downregulating miR-181a expression and activating the JNK and AMPK signaling pathways, suggesting that H19 exerts proangiogenic effects by regulating VEGF and eNOS [133]. The above experimental results show that H19 promotes angiogenesis under both physiological and pathological conditions.

Hypoxia-inducible factor 1α (HIF-1α) is a transcription factor that acts as a master regulator of oxygen homeostasis by regulating angiogenesis and glucose metabolism and plays a key protective role in the pathophysiology of pathological hypertrophy [134]. The ubiquitylation of HIF1α leads to a mismatch between myocardial growth and capillary density, thereby facilitating the development of maladaptive CH [135]. Intriguingly, in the nucleus of smooth muscle cells, accumulated H19 binds to the promoter region of HIF1α and recruits the transcription factor Sp1, which enhances HIF1α expression [93]. Whether H19 regulates HIF1α in cardiomyocytes is currently unclear.

These studies suggest that H19 regulates angiogenesis, which is a critical cause of CH. However, the specific mechanism underlying in myocardial hypertrophy needs further study.

## LncRNA-H19 regulates the inflammatory response

Studies have shown that H19 is involved in several kinds of inflammatory responses [136, 137], which are also involved in the pathogenesis of CH [138–140].

Some transcription factors of the inflammatory response, such as nuclear factor-κB (NF-κB), have been recognized to be related to the process of CH [141, 142]. Celecoxib, a classic anti-inflammatory agent, markedly prevents the expression of multiple inflammatory factors, including ICAM‐1, PAI‐1, and TNF‐α, in hypertrophic hearts *via* inhibition of the AKT/‐mTOR/NF‐κB signaling pathway [142]. When responding to inflammatory signals, NF-κB plays a pathogenic role in inflammation. NF-κB enhances interleukin 6 (IL-6) expression by downregulating microRNA let-7. A recent study in mice with TAC provided new insight into hypoxia-induced mitogenic factor (HIMF), a cytokine-like protein that can induce CH by regulating IL-6 [143].

In human umbilical vein endothelial cells (HUVECs), H19 depletion favors a pro-inflammatory environment characterized by IL-6 signaling and STAT3 activation [103]. Additionally, H19 can regulate inflammatory responses by directly targeting let-7. In the latest research, in a mouse model of abdominal aortic aneurysm (AAA), H19 overexpression in VSMCs increased IL-6 expression, promoted vascular inflammation, and ultimately induced AAA development [144]. This report suggests a possible H19/NF-κB/let-7/IL-6 pathway by which H19 regulates CH-related inflammatory responses.

## LncRNA-H19 regulates cellular apoptosis

In the normal heart, apoptosis occurs at rare low rates. However, in heart disease, this rate increases, which results in decompensated hypertrophy and HF, based on animal studies [145, 146]. Overexpression of H19 in vascular smooth muscle cells (VSMCs) and human umbilical vein endothelial cells (HUVECs) induces an increase in proliferation and a decrease in apoptosis [147]. Moreover, it has been reported that H19 promotes proliferation and inhibits apoptosis by modulating the WNT/β-catenin signaling pathway via miR-148b in ox-LDL-stimulated human aorta vascular smooth muscle cells (HA-VSMCs), suggesting that H19 may play a role in regulating cellular proliferation and apoptosis [148]. However, in a rat model of adriamycin-induced dilated cardiomyopathy, H19 was described to promote apoptosis [149]. Another study demonstrated that the H19/miR-675 axis is involved in regulating apoptosis by targeting voltage-dependent anion channel 1 (VDAC1) in cardiomyocytes exposed to high glucose [136]. As far as current research is concerned, H19 can positively or negatively regulate apoptosis, which may provide valuable insights for understanding the pathogenic role of H19 in the development of heart disease.

Through cell shrinkage, chromatin compaction, plasma membrane blebbing, and nuclear fragmentation, apoptosis plays critical roles in the pathogenesis of HCM [150]. The TAC mouse model results in hypertrophy with increased fibrosis, inflammation, cardiomyocyte apoptosis, and persistent CaMKIIδ activation [151]. However, the clear mechanism by which H19 regulates apoptosis in CH needs further study.

## LncRNA-H19 regulates cardiac remodeling post myocardial infarction

Myocardial infarction (MI) remains the leading cause of morbidity and mortality worldwide, despite significant progress in the treatment and prevention of the disease [152]. In addition to acute myocardial ischemic damage and reperfusion injury, heart failure triggered by the ensuing maladaptive ventricular remodeling can be a truly difficult issue to address [153].

Dynamic regulation of H19 post-MI is involved in multiple pathways of different cardiac cell types, including cardiomyocyte apoptosis and cardiac inflammation. Recently, aberrant expression of H19 has been detected in acute myocardial infarction (AMI) patients [154]. Intriguingly, Choong et al. observed that H19 is slowly upregulated and reaches an exceptionally high level and a significant increase in heart weight at day 4 post-MI [125]. Furthermore, the size of cardiomyocytes increased in H19-overexpressing mice at day 4 post-MI, suggesting that the overexpression of H19 indeed has an effect on cardiac hypertrophy. Further study found H19 competes with COL1A1 promoter to form the H19-YB-1 complex. The function of YB-1 as a suppressor of COL1A1 is abolished, and the expression of Col1a1 is increased and promotes the development of cardiac hypertrophy. In contrast, the study results were inconsistent with other studies, which concluded that H19 has antihypertrophic functions. Viereck et al. unraveled that H19 exerts its antihypertrophic functions by targeting the pro-hypertrophic nuclear factor of activated T cells (NFAT) signaling [68]. More recently, H19 could inhibit CYP1B1 expression in a PBX3-dependent manner to suppress cell apoptosis and promote cell proliferation, thus attenuating myocardial infarction [155]. Zhang and colleagues found that forced H19 expression could dramatically reduce myocardial infarction size, improve cardiac performance and alleviate cardiac fibrosis by mitigating myocardial apoptosis and decreasing inflammation [156]. Subsequent molecular mechanism experiments verified that H19 could function as an endogenous sponge to competitively bind to miR-22-3p to ameliorate MI-induced myocardial damage by upregulating the expression of KDM3A, which participated in left ventricular hypertrophy in response to pressure overload [157]. A potential explanation for this discrepancy is that lncRNAs can be differentially expressed in different cell types to exert distinct cell type-specific functions [22, 158].

As a result, H19 regulates cardiac remodeling through different mechanisms, such as transcriptional regulation, and serves as a microRNA sponge to inhibit microRNA function to attenuate myocardial infarction and MI-induced myocardial damage. However, further research is required to investigate whether there are other mechanisms that link H19 and the pathological process of AMI.

By genetic analysis, a recent study suggested a significant association between H19 gene variants and HCM [159], which requires validation in other large cohorts and functional studies to define the biological effect of these nucleotide changes. In addition, CH is also accompanied by changes in metabolism [160], oxidative stress [161], mitochondrial homeostasis [162], etc. However, whether H19 regulates these processes is still largely unknown, and the mechanism needs to be further elucidated.

## Conclusions

The goal of HCM therapy is to alleviate symptoms and prevent sudden death by the prohibition of competitive sports participation, septal alcohol ablation, septal myectomy, the implantation of cardioverter-defibrillators (ICDs) if needed, and cardiac transplantation [12]. With the elucidation of the underlying mechanism of pathological hypertrophy, many new perspectives for the targeted therapy of CH have been proposed. Mavacamten, named MYK-461, as an orally administered, small-molecule modulator of cardiac myosin, could selectively attenuate the activity of myosin ATPase to improve exercise capacity, left ventricular outflow tract obstruction, and health status in patients with obstructive hypertrophic cardiomyopathy [163].

Compared with most lncRNAs, H19 is a locus with a high degree of sequence conservation in mammals, which means that H19 has important functions and may be a potential therapeutic possibility as a targeting molecule in HCM.

Overexpression of H19 could reduce cardiomyocyte size in response to phenylephrine [67]. Moreover, to evaluate the effect of H19 on myocardium, Viereck et al. established a cardiomyocyte-specific H19 gene therapy approach [68]. These authors used cardiomyocyte-related adeno-associated virus 9 (AAV9) as a vector and the cardiomyocyte-specific TNNT2 promoter to inhibit H19 expression in cardiomyocytes. Interestingly, echocardiography assessment showed that both murine and human H19 overexpression stall hypertrophy progression. These findings indicate that H19 is highly conserved and dysregulated in hypertrophic hearts of pigs and humans. This emphasizes that H19 is a promising therapeutic target for pathological CH.

All these studies show that lncRNAs play a crucial role in the occurrence and progression of heart disease and will become a new hot spot and focus of cardiovascular basic and clinical research. Regulation of H19 expression provides a new direction for future treatment of CH. However, further research is warranted to clarify the following issues: (i) What processes regulates the H19 levels in the oncogenesis, development, and progression of CH? (ii) Accumulating data suggest that H19 is expressed in almost every human cancer [164, 165], so could H19 lead to unpredictable toxicity and side effects during the process of influencing CH? (iii) How does H19 participate in different pathways to regulate CH? (iv) Does H19 act on both myocardial cells and fibroblasts to regulate myocardial hypertrophy? (v) What are other H19 targets involved in regulating CH? Therefore, further research is needed to understand the functions of H19 that are widely involved in cardiac homeostasis, diseases and therapeutics, but toxic effects and other side effects must be considered.

## Outlook

Increasing evidence illustrated that H19, by acting as a molecular sponge or interacting with various proteins to regulate gene expression, could play an essential role in the complex network that regulates pathological hypertrophy progression, which includes intracellular calcium transition, fibrosis, angiogenesis, etc. These results indicate that H19 is a potential marker or a promising target for the treatment of CH. Nevertheless, there are still some questions to answer about the pathological mechanisms of CH. Thus, it is desirable to explore the molecular mechanisms and cellular pathways controlled by H19 in CH.

## Data Availability

Not applicable.
